# Spatial and Functional Aspects of ER-Golgi Rabs and Tethers

**DOI:** 10.3389/fcell.2016.00028

**Published:** 2016-04-18

**Authors:** Jaakko Saraste

**Affiliations:** Department of Biomedicine and Molecular Imaging Center, University of BergenBergen, Norway

**Keywords:** ER-Golgi transport, Golgi apparatus, pre-Golgi intermediate compartment, endocytic recycling compartment, Rab1, Rab2, golgins, tethering factors

## Abstract

Two conserved Rab GTPases, Rab1 and Rab2, play important roles in biosynthetic-secretory trafficking between the endoplasmic reticulum (ER) and the Golgi apparatus in mammalian cells. Both are expressed as two isoforms that regulate anterograde transport via the intermediate compartment (IC) to the Golgi, but are also required for transport in the retrograde direction. Moreover, Rab1 has been implicated in the formation of autophagosomes. Rab1 and Rab2 have numerous effectors or partners that function in membrane tethering, but also have other roles. These include the coiled-coil proteins p115, GM130, giantin, golgin-84, and GMAP-210, as well as the multisubunit COG (conserved oligomeric Golgi) and TRAPP (transport protein particle) tethering complexes. TRAPP also acts as the GTP exchange factor (GEF) in the activation of Rab1. According to the traditional view of the IC elements as motile, transient structures, the functions of the Rabs could take place at the two ends of the ER-Golgi itinerary, i.e., at ER exit sites (ERES) and/or *cis*-Golgi. However, there is considerable evidence for their specific association with the IC, including its recently identified pericentrosomal domain (pcIC), where many of the effectors turn out to be present, thus being able to exert their functions at the pre-Golgi level. The IC localization of these proteins is of particular interest based on the imaging of Rab1 dynamics, indicating that the IC is a stable organelle that bidirectionally communicates with the ER and Golgi, and is functionally linked to the endosomal system via the pcIC.

## Introduction

Yeasts genetics and biochemical dissection of cell free systems paved the way for the identification of the transport factors that mediate two-way trafficking between the endoplasmic reticulum (ER) and the Golgi apparatus in mammalian cells (Bonifacino and Glick, 2004; Lee et al., 2004). However, although the molecular machineries operating in the early secretory pathway—including coat proteins, Rab GTPases, tethering factors and SNAREs—have been well characterized, their subcellular sites of function in living cells remain only partially understood. One explanation is that our knowledge on the localization of these proteins is still largely based on light microscopy (LM), and ultrastructural data is in many cases limited or missing. On the other hand, the mapping of the homo- and heterotypic tethering and fusion events at the ER-Golgi boundary has been complicated by the recycling of the machinery proteins, resulting in interdependence of the antero- and retrograde pathways. Furthermore, the secretory system of the budding yeast *Saccharomyces cerevisiae*, in which many of the molecular players have been characterized, differs considerably from that of mammalian cells, as it consists of tubular networks, but lacks the characteristic Golgi stacks. However, whether yeast cells—or fungi and plants in general—are equipped with a distinct organelle, comparable to the intermediate compartment (IC) that operates in bidirectional trafficking between the ER exit sites (ERES) and the Golgi stacks in mammalian cells, remains an open question (Marie et al., 2008; Ito et al., 2012; Donohoe et al., 2013; Kurokawa et al., 2014).

Also, despite its well established role as a pre-Golgi sorting station in mammalian cells the IC remains enigmatic (Brandizzi and Barlowe, 2013; Saraste and Marie, 2016). Namely, contrasting with the popular view of the IC as a transient transport intermediate more recent studies employing live cell imaging have provided evidence for its stability and functional complexity (Ben-Tekaya et al., 2005, 2010; Sannerud et al., 2006). Moreover, they revealed the existence of a pericentrosomal subcompartment of the IC (pcIC), which is functionally connected with the centrosome and the endosomal system (Marie et al., 2009), providing a novel perspective to consider the spatial arrangement and function of the transport machineries operating in the early biosynthetic-secretory pathway (Saraste et al., 2009). Based on these results, this brief review addresses the functions of the IC-associated GTPases Rab1 and Rab2, as well as their partners that have been suggested to function in membrane tethering.

## Rab1 defines a novel pericentrosomal IC domain

As mentioned above, the IC is commonly thought to consist of tubulovesicular membrane clusters that form *de novo* at ERES, move along MTs to the *cis*-Golgi and—depending on whether the Golgi is regarded as a maturing or stationary organelle—transform into new Golgi cisternae or fuse with the Golgi stacks (Brandizzi and Barlowe, 2013; Saraste and Marie, 2016). The alternative view of the IC as a stable compartment derives mainly from studies on the dynamics of fluorescent IC markers in living cells. First, visualization of p58/ERGIC-53, a cargo-receptor that cycles at the ER-Golgi interface, showed its presence in long-lived, relatively stationary structures close to ERES (Ben-Takaya et al., 2005; Sannerud et al., 2006). Second, the employment of the GTPase Rab1A as a specific IC marker revealed a dynamic network of interconnected tubular and saccular elements operating between the peripheral ERES and the central Golgi apparatus. However, instead of moving directly to the *cis*-Golgi, the pleiomorphic carriers arriving from the ERES are first targeted to a distinct domain of the IC that associates with the centrosome, termed the pericentrosomal IC (pcIC) (Marie et al., 2009). Notably, the pcIC is a stable compartment, which maintains its pericentrosomal positioning and compositional properties, when the Golgi apparatus is experimentally broken down by the fungal compound brefeldin A (BFA) (Marie et al., 2009; Mochizuki et al., 2013), or undergoes physiological disassembly during mitosis (Marie et al., 2012).

**Figure 1:**
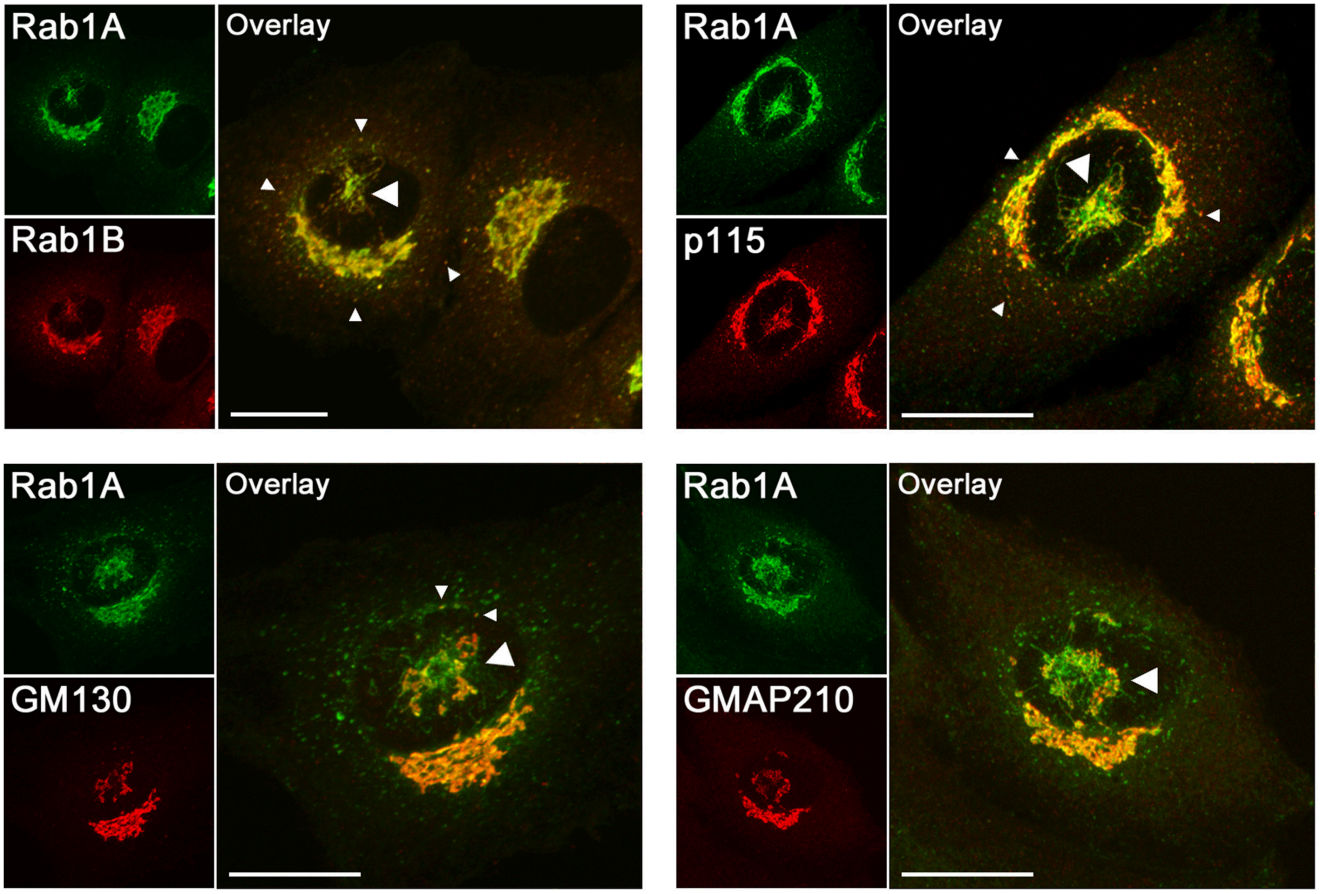
**Confocal microscopic localization of the two Rab1 isoforms and the effectors of Rab1 (p115, GM130) and Rab2 (GM130, GMAP210) that have been suggested to function in membrane tethering in the early secretory pathway**. Normal rat kidney (NRK) cells stably expressing GFP-Rab1A (Marie et al., 2009) were stained with antibodies against Rab1B, p115, GM130, or GMAP210. The images show cells in which the pcIC, an extensive tubular network under the nucleus (large arrowheads), has separated from the Golgi ribbon concomitantly with the movement of the centrosome to the cell center. Rab1A and B display similar localizations; that is, in addition to the Golgi ribbon they both associate with the pcIC, as well as with peripheral IC elements in the vicinity of ERES (small arrowheads). It should be noted that Rab1A and the tethering factors display variable overlap in the pcIC, suggesting their association with its different subdomains. Bars: 10 μm.

Fortunately, while the pcIC during interphase is concealed by the Golgi ribbon, it separates from the latter when cells start to move or enter mitosis—i.e., events that involve centrosome motility, resulting in Golgi repositioning or complete disassembly (Bisel et al., 2008; Marie et al., 2012). This separation (see Figure 1) allowed the demonstration of its function as the primary target for the pleiomorphic cargo carriers that originate at peripheral ERES and move to the cell center along MTs (Marie et al., 2009). In addition, the separated pcIC maintains two-way communication with the Golgi apparatus via tubular and vesicular carriers, and operates as a way station in BFA-stimulated tubular transport of Golgi enzymes to the ER (Marie et al., 2009). Thus, bidirectional trafficking via the pcIC may involve both COPI-dependent and -independent mechanisms.

**Figure 2:**
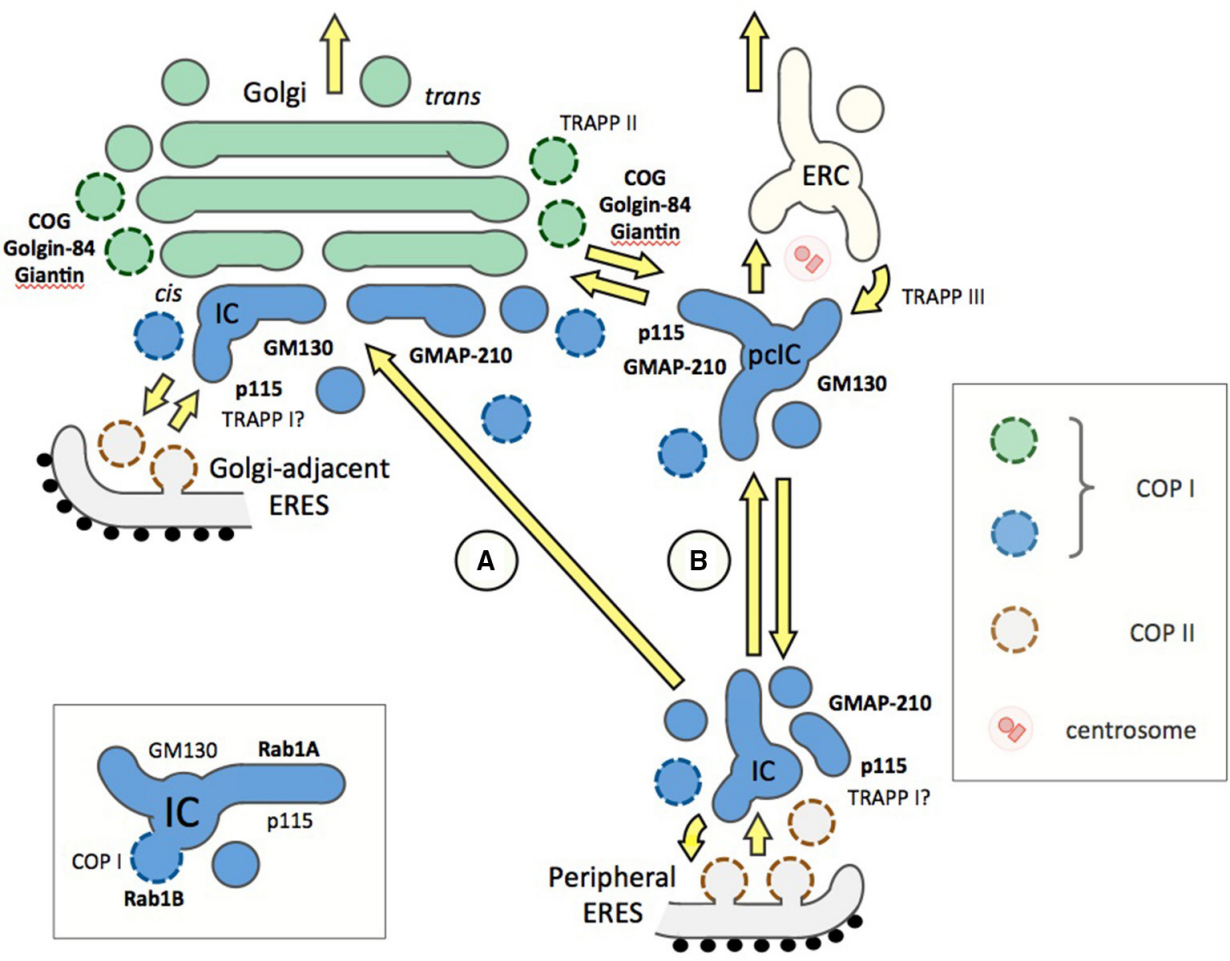
**Different models on the organization of the ER-Golgi interface and the functions of Rab1 or Rab2 effectors (bold) or Rab1 GEFs (TRAPPs) in trafficking**. Two alternative pathways connecting the peripheral ERES and the Golgi stacks (green) are shown (A and B), while Golgi-adjacent ERES is a common feature of both models. The pcIC is depicted as a separate entity (see Figure 1), since its normal dynamic relationship with the Golgi apparatus remains unknown. At ERES, homotypic fusion of ER-derived COPII vesicles or their heterotypic fusion with the IC elements involve TRAPPI and p115, the GEF, and effector of Rab1, respectively. However, it should be noted that the TRAPPI complex in mammalian cells remains enigmatic. (A) Traditionally, IC-to-Golgi transport is viewed as a one-step process based on MT-based direct movement of IC elements from peripheral ERES to the *cis*-Golgi region, where they undergo homotypic fusion to generate new Golgi cisternae in a process that may involve e.g., GM130, an effector of both Rab1 and Rab2. Instead of operating in IC or *cis*-Golgi events many of the Rab1 effectors (such as COG, giantin, and golgin-84) could play a role in intra-Golgi trafficking. (B) An alternative model presenting the IC as a dynamic membrane network, which is stably anchored next to the centrosome. Accordingly, transport from peripheral ERES to the Golgi is a two-step process via the pcIC, opening for possible new roles for Rab1, Rab2 and their effectors. These include homotypic fusion of pcIC elements (GM130), retrograde transport from the pcIC to peripheral ERES (GMAP-210), and two-way trafficking between the pcIC and the Golgi stacks (p115, giantin, golgin-84, COG), as well as between pcIC and the ERC (TRAPPIII). The Rab1 isoforms are expected to be present throughout the IC network (blue), while the localization of the Rab2 isoforms is less well known. For simplicity, the endosomal connection is not included in model A. The inset (bottom left) depicts a basic IC element, showing differential association of Rab1A and Rab1B with its tubular and saccular (vacuolar) membrane domains, raising the possibility that Rab1 effectors, such as p115 and GM130, may display non-overlapping distributions within these elements.

The function of the pcIC as a central trafficking “hub” that is independent from the Golgi stacks is also demonstrated by its close relationship with the endocytic recycling compart-ment (ERC) (Figure 2). Namely, the spatial connection of these compartments, defined by Rab1 and Rab11, respectively, is maintained after Golgi disassembly by BFA and allows the direct exchange (“Golgi bypass”) of certain newly synthesized molecules and internalized plasma membrane receptors between the IC and the endosomal system (Saraste et al., 2009). For example, when the normal recycling of the transferrin receptor from the ERC to the plasma membrane blocked in the presence of BFA, it can return to the cell surface via the pcIC (Marie et al., 2009).

In conclusion, due to its division into vacuolar (saccular) and tubular subdomains, spatial complexity and connection with the centrosome, the IC shares striking similarity with the endosomal system (Saraste and Goud, 2007; Saraste et al., 2009). Thus, many of the Rab proteins that function at the ER-Golgi boundary (Gilchrist et al., 2006; Liu and Storrie, 2015), such as Rab1 and Rab2, could be expected to have organizational roles resembling those that have been well established for the endosomal Rabs (Wandinger-Ness and Zerial, 2014).

## IC localization and function of Rab1 and Rab2

Rab GTPases coordinate multiple steps of transport along the secretory and endocytic pathways, including the formation, motility, tethering and fusion of vesicular, and tubular transport intermediates. By switching between inactive, GDP-bound (cytosolic) and active, GTP-bound (membrane-associated) states they are thought to function as master regulators of membrane trafficking and to ensure the directionality of transport (Stenmark, 2009). In their active conformation Rab proteins interact with various effectors and recruit these to specific membrane domains, thereby also determining organelle structure and identity (Barr, 2013). The functions of multiple Rabs acting along the same pathway can be connected via mechanisms involving e.g., GTP exchange factors (GEFs) or GTPase-activating proteins (GAPs) that regulate the switch between the active and inactive forms, respectively. An example is provided by the Ypt1(Rab1)-Ypt31/32(Rab11)-Sec4(Rab8) cascade that co-ordinates the secretory pathway in the budding yeast (Mizuno-Yamasaki et al., 2012; Lipatova et al., 2015).

The best characterized Rabs operating in ER-Golgi trafficking in mammalian cells, Rab1 and Rab2, are both expressed as two isoforms (A and B) that share a high degree of sequence similarity (~92 and 86%, respectively). They were first shown to function in anterograde ER-Golgi transport and suggested to operate in a sequential manner (Tisdale et al., 1992). However, subsequent studies have shown that they—like Ypt1 in yeast (Kamena et al., 2008)—are also required for retrograde transport. Rab1 has been localized to retrograde IC tubules (Palokangas et al., 1998; Sannerud et al., 2003; Marie et al., 2009), while both Rab1 and Rab2 regulate the binding of COPI coats to IC membranes (Tisdale and Jackson, 1998; Alvarez et al., 2003). Of note, although COPI coats function in retrograde trafficking, a recent study re-emphasized their additional role in anterograde transport at the Golgi level (Park et al., 2015). Finally, an LM-based screen identified the Rab1 and Rab2 isoforms as key regulators of retrograde transport of Golgi enzymes to the ER in BFA-treated cells (Galea et al., 2015), further demonstrating their requirement for bidirectional ER-Golgi communication.

Besides ER-Golgi trafficking, the isoforms of Rab1 and Rab2 are also important for Golgi biogenesis (Wilson et al., 1994; Haas et al., 2007; Rendón et al., 2013; Liu and Storrie, 2015), indicating that the two prosesses are intimately coupled. Accordingly, their knock-down results in the fragmentation of the Golgi ribbon (Galea et al., 2015). Interestingly, a recent study showed that the Golgi fragmentation phenotype could be induced by single knock-down of each of the four isoforms. Moreover, normal Golgi organization could only be rescued by the re-expression of the same isoform, but not by any of the others, suggesting that the isoforms have non-redundant functions (Aizawa and Fukuda, 2015).

There is also evidence indicating that the Rab1 isoforms associate with different domains of the IC and regulate distinct steps of trafficking. By live cell imaging Rab1A highlights dynamic IC tubules that display microtubule (MT)-dependent, bidirectional movements throughout the cytoplasm (Sannerud et al., 2006; Marie et al., 2009), while Rab1B appears to preferentially localize to more stationary, punctate IC structures (Monetta et al., 2007). These may correspond to the saccular (vacuolar) IC elements where Rab1B interacts with GBF1, a GEF for ARF1, which regulates the budding of COPI vesicles (Alvarez et al., 2003; Figure 2, inset). Notably, the entry of cells into mitosis results in the cessation of tubular IC dynamics, but not the budding of IC-derived COPI vesicles (Marie et al., 2012), which could be due to differential phosphorylation of the two Rab1 isoforms (Bailly et al., 1991). However, it should be noted that Rab2 which also acts in COPI recruitment is not subjected to mitotic phosphorylation (Bailly et al., 1991).

Initial *in vitro* studies suggested that Rab1 also mediates intra-Golgi transport (Plutner et al., 1991). However, subsequent studies using electron microscopy (EM) showed that the Golgi-type signal seen by LM (Figure 1) is not due to the presence of Rab1 in the Golgi cisternae themselves, but results from the co-alignment of pleiomorphic IC elements along the *cis*-face of the Golgi stacks (Griffiths et al., 1994; Saraste et al., 1995; Satoh et al., 2003; Marie et al., 2012). These EM studies and cell fractionation experiments (Sannerud et al., 2006) have also clarified that Rab1 does not associate with the ER (see Figure 1). Thus, these collective results do not support the frequent assignment of the Rab1 isoforms as ER or Golgi proteins, but establish them as specific markers of the IC. The recently established localization of Rab1A and Rab1B to the pcIC (Marie et al., 2009, 2012; Mochizuki et al., 2013; Figure 1) is in accordance with this conclusion. Moreover, it opens the possibility that, besides peripheral IC elements (Sannerud et al., 2006; Monetta et al., 2007), these Rab1 isoforms are also recruited to the pcIC and may play distinct roles in its bidirectional communication with the Golgi stacks or the ERC (Figure 2).

Although Rab2 was the first family member shown to associate with the IC (Chavrier et al., 1990; Lotti et al., 1992), and to mediate the budding of COPI vesicles (Tisdale and Jackson, 1998), its overall localization has not been well characterized. Also, the dynamics of Rab2 has not been recorded in living cells. Interestingly, however, Rab2A was recently shown by LM to overlap with ERGIC-53 and Rab1B (Sugawara et al., 2014; Galea et al., 2015), confirming its IC localization.

## The partners of Rab1 and Rab2

Most of the well-characterized effectors of Rab1 and Rab2 belong to the golgin family of peripheral or integral membrane proteins that based on their C-terminal anchoring and elongated shape (long coiled-coil domains) are capable of tethering membranes prior to their eventual fusion (Munro, 2011; Chia and Gleeson, 2014). As the name implies, the golgins have been assigned various Golgi-specific functions. Originally suggested to form a dense matrix supporting the integrity of the Golgi stacks, they have subsequently been implicated in the linking of the Golgi ribbon (Puthenveedu et al., 2006; reviewed by Xiang and Wang, 2011). Furthermore, the specific localization of golgins to different Golgi domains, and their overlapping abilities to bind multiple Rabs along their length were proposed to endow the Golgi stacks with “tentacles” that collectively capture incoming transport vesicles (Munro, 2011; Wong and Munro, 2014).

The first identified Rab1 effector was p115, a myosin-like coiled-coil protein that interacts with several other transport factors and COPI coats and appears to play multiple roles in bidirectional ER-Golgi trafficking and Golgi biogenesis (Alvarez et al., 1999; Allan et al., 2000; Sztul and Lupashin, 2009). The other partners of Rab1 include the prototype golgin GM130, as well the integral membrane proteins giantin and golgin-84 (Moyer et al., 2001; Weide et al., 2001; Diao et al., 2003; Beard et al., 2005; Rosing et al., 2007), which besides linking COPI vesicles to Golgi membranes may also participate in other types of tethering events (Malsam et al., 2005; Wong and Munro, 2014). In addition to GM130, Rab2 interacts with the golgin GMAP-210 (Short et al., 2001; Sinka et al., 2008; Sato et al., 2015), whose tethering function is based on its ability to sense membrane curvature (Drin et al., 2008). Via their interactions with golgin-45 and GM130, respectively, Rab2 and Rab1 may also be linked to GRASP55 and GRASP65, which play key roles in Golgi organization (Short et al., 2001; Xiang and Wang, 2011).

Notably, besides membrane tethering, the golgins are also involved in membrane-cytoskeleton interactions. Thus, GMAP-210 has been suggested to link Golgi membranes to the centrosome-nucleated MT cytoskeleton (Rios et al., 2004), while GM130 participates in the nucleation of MTs from *cis*-Golgi membranes (Rivero et al., 2009).

Rab1 and Rab2 also interact with multi-subunit tethering complexes, such as the COG complex that organizes COPI vesicle-mediated recycling of Golgi enzymes (Willett et al., 2013) and TRAPP, which also functions as a GEF for Ypt1/Rab1 (Barrowman et al., 2010). In yeast the TRAPP complex exists as three forms (I-III) of which at least TRAPPII and III seem to have mammalian counterparts (Yamasaki et al., 2009; Scrivens et al., 2011; Bassik et al., 2013; Lamb et al., 2016). TRAPPI and II function successively in early and late Golgi trafficking in the yeast secretory pathway, while TRAPPIII activates Ypt1 during autophagy (Barrowman et al., 2010; Lipatova et al., 2015). Similarly, Rab1 has been shown to regulate the formation of autophagosomes in mammalian cells (Winslow et al., 2010; Zoppino et al., 2010; Huang et al., 2011; Mochizuki et al., 2013; Lamb et al., 2016).

## Pre-golgi roles of Rabs and tethers

Based on the traditional view of the IC as transient vesicular-tubular clusters (VTCs), anterograde transport from ERES to *cis*-Golgi could represent a one-step process. Accordingly, Rab1, Rab2, and their partners could exert their functions at the two ends of the MT-dependent pathway, i.e., during the formation of the IC elements at ERES and/or their homo- or hetero-typic fusion at *cis*-Golgi. In addition, some of the Rabs would be required for membrane recycling from IC/*cis*-Golgi back to the ER. According to this view (Figure 2; *model A*), multiple tethering factors could operate in a sequential—or possibly redundant—fashion at the same transport step. It has been suggested that an additional role of the tethers is to couple antero- and retrograde trafficking (Sztul and Lupashin, 2009).

By contrast, taking into account the stable connection of the pcIC with the centrosome, the communication between ERES and *cis*-Golgi is expected to involve two transport steps. Accordingly, the IC elements arriving from the peripheral ERES first fuse with the pcIC, which most likely represents a dynamic membrane system normally located close to the Golgi ribbon. Consequently, a second transport step would be required for trafficking between the pcIC and the Golgi stacks. This new model is supported by the observed localization the Rab1 isoforms and COPI coats to the pcIC (Marie et al., 2009; Mochizuki et al., 2013). Interestingly, many of the Rab1 and Rab2 effectors are present in the pcIC, as shown for p115, GM130, and GMAP-210 in Figure 1. Thus, they could participate in two-way trafficking between this compartment and the Golgi stacks, or even be required for its communication with the ERC (Figure 2, *model B*). The localization of GM130 and GMAP210 to the pcIC (Figure 1) also provides indirect proof for the presence of Rab2 itself in this compartment.

Notably, the pcIC localization of these Rabs and their effectors, such as p115, GM130, giantin and golgin-84, persists in BFA-treated cells (Seemann et al., 2000; Steet and Kornfeld, 2006; Marie et al., 2009; Mochizuki et al., 2013; Roboti et al., 2015), showing their specific association with the IC. Moreover, during mitosis, when ER-Golgi transport is inhibited and the Golgi undergoes reversible disassembly, these proteins maintain their association with the pcIC membranes at the spindle poles (Marie et al., 2012).

The IC localization of many of the golgins has already been shown previously. Although first implicated in intra-Golgi transport, p115 was subsequently shown to associate with peripheral IC elements and function at an early stage of ER-Golgi trafficking (Alvarez et al., 1999, 2001; Allan et al., 2000). Moreover, immuno-EM detected p115 in pleiomorphic, tubulovesicular elements at the *cis*-face of the Golgi stacks (Nelson et al., 1998), most likely corresponding to the IC elements that also harbor Rab1 (Marie et al., 2012). Similarly, there is additional evidence showing that the other golgins are not restricted to the vicinity of the Golgi apparatus, but also associate with the IC and operate in ER-Golgi trafficking. For example, GM130 has been localized to IC elements and suggested to function in their homotypic fusion to generate the Golgi ribbon (Marra et al., 2001, 2007). Notably, GM130 preferentially associates with tubular networks at the *cis*-face of the mammalian Golgi ribbon or the separate Golgi stacks of *Drosophila* cells (Martínez-Alonso et al., 2005; Sinka et al., 2008; Vivero-Salmerón et al., 2008), rather than the Golgi cisternae. Recently, GMAP210 was localized to the IC and shown to be required for multiple antero- and retrograde transport steps at the ER-Golgi boundary. Interestingly, experiments with BFA indicated that its depletion blocks retrograde Golgi-to-ER transport at the level of the drug-resistant pcIC (Roboti et al., 2015).

Thus, the golgins could be involved in the multiple transport steps at the ER-Golgi interface. Besides acting in membrane tethering and fusion processes at ERES, they could participate in transport events that take place between the peripheral IC elements and the pcIC, and/or function in pcIC-Golgi trafficking (Figure 2, *model B*). This new scenario raises the possibility that the COPI vesicles in the vicinity of the Golgi apparatus—defined by the tethers golgin-84 and p115 (Malsam et al., 2005), and proposed to function in both antero- and retrograde intra-Golgi trafficking (Orci et al., 1997)—could instead mediate transport between the pcIC and the Golgi stacks. Furthermore, the persistent association of GM130 and p115 with the pcIC during the cell cycle (Marie et al., 2012) could explain their effects on the organization of the centrosome and the mitotic spindle (Kodani et al., 2009; Radulescu et al., 2011).

Regarding the multi-subunit tethers, COG has been shown to associate with tubulo-vesicular clusters resembling the IC in the vicinity of the Golgi stacks (Vasile et al., 2006) and influence trafficking at the ER-Golgi boundary (Steet and Kornfeld, 2006). Subunits of the mammalian TRAPP complexes co-localize with IC/*cis*-Golgi markers p58/ERGIC-53, GM130, and COPI, and their knock-down seemed to arrest anterograde transport at the level of the peripheral IC elements (Yamasaki et al., 2009; Scrivens et al., 2011). Moreover, the common TRAPP subunit mBet3 has been detected in BFA-resistant structures resembling the pcIC (Yu et al., 2006). Notably, it was recently shown that the mammalian TRAPPIII complex links the functions of Rab11 and Rab1 in the delivery of membranes from the ERC to forming autophagosomes, providing evidence for its role in constitutive trafficking between the pcIC and the ERC (Lamb et al., 2016).

## Summary and perspectives

Imaging of Rab1 dynamics in living cells uncovered a novel spatial aspect of ER-Golgi communication by showing the permanent anchoring of the dynamic IC network to the centrosome. The pcIC clearly represents a specialized compartment distinct from the Golgi stacks, as shown by its BFA-resistant nature, division at the onset of mitosis, and communication with the ERC. However, as this compartment reveals itself only under special circumstances, a major challenge for the future is to clarify its relationship with the traditional Golgi system. In light of relevant literature I have explored here the possibility that the functional landscape of the primary ER-Golgi Rabs and their “tethering partners” could be more complex than previously anticipated, taking into consideration the stable nature of the pcIC and its functional connection with the centrosome and the endosomal recycling system. Besides identifying its transport machineries, and its role in Rab activation, future studies could provide important information on this pericentrosomal membrane system by addressing its non-trafficking roles.

## Author contributions

The author confirms being the sole contributor of this work and approved it for publication.

## Funding

This work was supported by the Nansen Foundation and the Aurora Program of the Norwegian Research Council (244125/O30).

### Conflict of interest statement

The author declares that the research was conducted in the absence of any commercial or financial relationships that could be construed as a potential conflict of interest.
